# General Commentary on: Alternatives to Antibiotic Growth Promoters in Animals

**DOI:** 10.3389/fvets.2016.00074

**Published:** 2016-08-31

**Authors:** Blessing O. Anonye

**Affiliations:** ^1^Microbiology and Infection Unit, Division of Biomedical Sciences, Warwick Medical School, University of Warwick, Coventry, UK

**Keywords:** antibiotics growth promoters, phytogenic feed additives, microbiota, antibacterial activity, probiotics

Recent studies have evaluated the impact of alternatives to antibiotic growth promoters (AGPs) such as phytogenic feed additives (PFAs) *in vitro* and *in vivo*. Zhou and colleagues studied the antibacterial properties of 30 herbs on pathogenic Gram-negative and positive bacteria (1). Thirteen of the 30 herbs exerted a significant effect against *Escherichia coli* ATCC 25922 (*p* < 0.01, *n* = 11 and *p* < 0.05, *n* = 2). More than 30% of the herbs exhibited activity against *Salmonella enteritidis* ATCC 13076 and *Salmonella typhimurium* ATCC 14028. These pathogenic organisms commonly infect humans and animals especially poultry such as chickens leading to loss and decrease in their market value. Additionally, Zhou reported that more than 50% of the herbal extracts (*n* = 16) possessed antibacterial activity against *Staphylococcus aureus* ATCC 25923.

Furthermore, based on the results of the individual herbs on the pathogenic bacteria, two formulations were performed with five of the herbs. For formulation 1, the herb, *Fructus mume* was the main ingredient (35%) and *Galla chinensis* (30%) for formulation 2 with varying proportions of four other herbs. Both formulations 1 and 2 had significant antibacterial activity against the pathogenic bacteria (*p* < 0.05) with no significant difference in activity when compared to the AGPs, aureomycin, and flavomycin (1). Formulation 1 also led to increased counts of *Lactobacillus acidophilus* ATCC 4356 and *Bifidobacterium longum* ATCC 15707 relative to the control, indicating the possibility that these herbs could have a synergistic effect on beneficial bacteria in the intestinal microbiota. This raises the potential of using herbs as an alternative to antibiotics to increase growth in animals and modulate the microbiota. However, these herbs must be carefully chosen as formulation 2 did not produce the same probiotic effects as formulation 1 but led to reduced amounts of the *L. acidophilus* and *B. longum* compared to the control.

Similarly, Murugesan et al. compared the effects of Digestarom^®^ Poultry, a commercial PFA produced by BIOMIN, to the AGP, bacitracin methylene disalicylate in broiler chickens (2). Chicks were randomly assigned to receive either a corn–soybean meal only or supplemented with the PFA or AGP, respectively, over a 39-day period. This period was divided into pre-starter (days 1–7), starter (days 8–21), and grower (days 22–39) phases. The authors noted differences based on the period of growth. For example, in the starter phase, AGP-fed birds gained more body weight relative to control, while PFA-fed birds had increased body weight in the grower phase. Also, increase in the villus height across the small intestine was observed in birds fed with AGP or PFA relative to control (2). As the villi help to increase the surface area of the intestinal walls, an increase in digestion and absorption of nutrients is likely to be observed (3). Coliforms were significantly decreased (*p* < 0.01), and *Lactobacillus* spp. was significantly increased (*p* < 0.01) through plating of the cecal microbiota when compared to the control or AGP-fed birds. Similar results have been obtained with respect to increased *Lactobacillus* spp. in PFA-fed birds using similar PFA as Murugesan et al. (4) or a phytoncide (5). However, next-generation sequencing could provide a better picture of the changes taking place in the cecal microbiota with respect to the bacterial groups.

The results from these and other studies suggest the ability of the PFAs to modulate the intestinal microbiota. These could occur through various mechanisms by influencing the digestibility of nutrients and thereby enhancing the growth performance of the animals (1, 2, 5). PFAs can potentially stimulate the secretion of digestive enzymes, thereby promoting gut functions. Moreover, the bioactive compounds produced by the PFAs have been shown to possess antibacterial properties *in vivo* against chickens challenged with *S. enteritidis, E. coli*, and *Clostridium perfringens* (4, 6). PFAs, such as *F. mume*, may exert their antibacterial effect through the production of organic acids, leading to increased acidity as revealed by HPLC (7). Another possible mechanism by which PFAs exert their beneficial effects is by acting as antioxidant against oxidative stress in animals.

However, the search for PFAs with these desirable properties is not trivial. Single and different combinations of PFAs need to be tested against different strains of pathogenic bacteria *in vitro* and *in vivo* to determine their antimicrobial activity. A desirable PFA ideally should be able to stimulate the gut microbiota in a number of ways. This could be through increasing colonization resistance without having any adverse effect on beneficial bacteria and creating a favorable environment for increased nutrient intake leading to weight gain. Both studies described above have shown to some extent these desirable characteristics, but more studies will be needed to determine the exact mode of action of these PFAs.

It would also be worth looking at other alternatives to antibiotics such as prebiotics, probiotics, and bacteriocins to determine their effects on animals (8, 9). The development of PFAs and other substances that can give similar or more beneficial outcomes, as the AGPs will go a long way in reducing the increase of antibiotic-resistant bacteria.

## Author Contributions

The author confirms being the sole contributor of this work and approved it for publication.

## Conflict of Interest Statement

The author declares that the research was conducted in the absence of any commercial or financial relationships that could be construed as a potential conflict of interest.
